# Dexborneol Amplifies Pregabalin’s Analgesic Effect in Mouse Models of Peripheral Nerve Injury and Incisional Pain

**DOI:** 10.3390/antiox13070803

**Published:** 2024-07-02

**Authors:** Zhen Shen, Yun-Dan Guo, Ming-Ze Tang, Ping Zhou, Yu-Xin Su, Hao-Ran Shen, Tao Li, Wei Jiang, Yan-Xing Han, Cai Tie, Jing-Jing Cui, Tian-Le Gao, Jian-Dong Jiang

**Affiliations:** 1 Institute of Materia Medica, Chinese Academy of Medical Sciences, Peking Union Medical College, Beijing 100050, China; ab11228866@163.com (Z.S.); guoyundan1011@163.com (Y.-D.G.); tangmz1998@163.com (M.-Z.T.); shenhaoran@imm.ac.cn (H.-R.S.); hanyanxing@imm.ac.cn (Y.-X.H.); jiang.jdong@163.com (J.-D.J.); 2 Heart Failure Center, Fuwai Hospital, National Center for Cardiovascular Diseases, Chinese Academy of Medical Sciences, Peking Union Medical College, Beijing 100037, China; zhoupingfw@sina.com; 3 Institute of Acupuncture and Moxibustion, China Academy of Chinese Medical Sciences, Beijing 100700, China; syxznzn@126.com; 4 Beijing Key Laboratory of Traditional Chinese Medicine Basic Research on Prevention and Treatment of Major Diseases, Experimental Research Center, China Academy of Chinese Medical Sciences, Beijing 100700, China; hndxlitao@163.com; 5 Zhejiang Zhenyuan Pharmaceutical Co., Ltd., Shaoxing 312071, China; zyrd05@zypharm.com; 6 State Key Laboratory for Fine Exploration and Intelligent Development of Coal Resources, School of Chemical and Environmental Engineering, China University of Mining and Technology (Beijing), Beijing 100083, China; 7 Institute of Medicinal Biotechnology, Chinese Academy of Medical Science, Beijing 100050, China

**Keywords:** chronic pain, combination therapy, neuroinflammation, HMGB1, ATP, Nrf2

## Abstract

Pregabalin is a medication primarily used in the treatment of neuropathic pain and anxiety disorders, owing to its gabapentinoid properties. Pregabalin monotherapy faces limitations due to its variable efficacy and dose-dependent adverse reactions. In this study, we conducted a comprehensive investigation into the potentiation of pregabalin’s analgesic effects by dexborneol, a neuroprotective bicyclic monoterpenoid compound. We performed animal experiments where pain models were induced using two methods: peripheral nerve injury, involving axotomy and ligation of the tibial and common peroneal nerves, and incisional pain through a longitudinal incision in the hind paw, while employing a multifaceted methodology that integrates behavioral pharmacology, molecular biology, neuromorphology, and lipidomics to delve into the mechanisms behind this potentiation. Dexborneol was found to enhance pregabalin’s efficacy by promoting its transportation to the central nervous system, disrupting self-amplifying vicious cycles via the reduction of HMGB1 and ATP release, and exerting significant anti-oxidative effects through modulation of central lipid metabolism. This combination therapy not only boosted pregabalin’s analgesic property but also notably decreased its side effects. Moreover, this therapeutic cocktail exceeded basic pain relief, effectively reducing neuroinflammation and glial cell activation—key factors contributing to persistent and chronic pain. This study paves the way for more tolerable and effective analgesic options, highlighting the potential of dexborneol as an adjuvant to pregabalin therapy.

## 1. Introduction

Pain is a distressing experience that is associated with actual or potential tissue damage, and is also influenced by factors including sensation, emotion, cognition, and social relationships [1]. Chronic pain is defined as persistent pain lasting over half a month, often stemming from conditions such as nerve damage, pulmonary infection, diabetes, liver disease, kidney disease, or multiple sclerosis. In China, chronic pain affects approximately 400 million patients. However, less than 60% of patients proactively seek treatment, and only 20% of those treated receive adequate pain relief [2]. According to the data from the Centers for Disease Control and Prevention (CDC), roughly a third of Americans have experienced chronic pain, resulting in staggering economic losses upwards of several hundred billion dollars per year due to medical expenses and decreased productivity.

The pathological mechanisms behind chronic pain are not fully understood but involve factors like inflammation, oxidative stress, glial cell activation, and neurotransmitter imbalances [3]. Oxidative stress, in particular, plays a pivotal role. It disrupts nervous system functions, altering pain perception and transmission, potentially leading to hyperalgesia [4]. Oxidative stress weakens the body’s inherent antioxidant protections, intensifying inflammation and harming cells and tissues. This leads to a destructive cycle: oxidative stress feeds chronic pain, which then generates more oxidative stress [5]. Grasping these dynamics is essential for developing effective treatments for chronic pain.

Currently, chronic pain treatment primarily depends on GABAergic anticonvulsants and opioids, but their efficacy remains limited. Anticonvulsants like gabapentin and pregabalin are often used as frontline therapies for chronic pain. These analgesics, acting as γ-aminobutyric acid (GABA) receptor agonists, specifically target the type I α2-δ subunit of voltage-dependent calcium channels in the pain-relaying networks. This targeted binding diminishes calcium ion influx, lowers excitability, and curtails neurotransmitter release, efficiently addressing neuropathic pain [6]. However, their effectiveness has been reported as less than satisfactory in clinical settings. Specifically, in traumatic nerve injury cases, these medications provide significant pain relief to less than half of the patients [7]. For mixed neuralgia, only 21% of patients achieve notable relief [8], and in band neuralgia, just 32–34% of patients report the drug’s effectiveness [9]. In recent years, reports of neurological and systemic adverse reactions to gabapentin and pregabalin have been on the rise. Clinical research suggests that these side effects, which include drowsiness, dizziness, peripheral edema, fatigue, and visual impairment, are positively associated with the applied doses [10]. On the other hand, opioids, which are traditional analgesics, despite exerting potent analgesic effects, are constrained by their severe side effects such as tolerance, dependence, and addiction [11]. 

Facing the constraints of present chronic pain treatments, we are seeking alternative therapeutic approaches. Recent progress in combined medication offers promise in overcoming the limits of current analgesics, with studies showing that a large portion of chronic pain patients find better pain relief with combinational therapy [12]. Our priority is identifying drug combinations that boost the potency of gabapentin or pregabalin while minimizing doses to reduce their side effects. In this regard, we have turned our focus to natural compounds derived from various natural sources, such as herbs and microorganisms; these compounds often exhibit multiple therapeutic effects, as revealed by animal studies [13,14]. However, while offering significant therapeutic potential, they also possess complex actions and possibly unknown primary targets. Despite this, they coexist harmoniously with humans and effectively treat chronic diseases, partly by regulating the gut microbiota [15]. Our laboratory’s successful demonstration of enhanced analgesic effects by combining sinomenine/ligustrazine with gabapentin in experimental pain models aligns with the goal of improving patients’ overall quality of life [16,17].

Dexborneol is a neuroprotective bicyclic monoterpenoid derived from herbs like *Cinnamomum camphora*. Its unique chemical structure gives dexborneol its notable pharmacological properties, including blood–brain barrier modulation, oxidative stress reduction, and inflammation suppression. Dexborneol’s antioxidation mechanism lies in its capacity to neutralize free radicals and reactive oxygen species (ROS) [18]. Based on previous animal research, dexborneol has the potential to preserve cellular integrity and halt the development of chronic pain associated with oxidative damage by decreasing oxidative stress [19]. Moreover, dexborneol fulfills the courier herb role in traditional Chinese medicine (TCM)’s monarch, minister, assistant, courier paradigm. This paradigm is crucial to TCM, combining herbs holistically for best therapy. The monarch targets the main issue, the minister aids or treats secondary symptoms, the assistant lessens side effects, and the courier, like dexborneol, guides herbs to targets, enhancing effectiveness. Dexborneol is seldom administered alone; instead, it potentiates the therapeutic benefits of accompanying medications when administered concurrently. Uniquely, it has the ability to steer medicinal agents upward, facilitating targeted drug delivery to the central nervous system. Preclinical research reveals that dexborneol promotes the penetration of drugs across the blood–brain barrier, thereby extending the effective residence duration of the administered drugs [20].

The aim of this study is to verify whether dexborneol can amplify the analgesic effect of pregabalin in animal pain models induced by peripheral nerve injury or incision. Furthermore, the study seeks to decipher the possible synergistic analgesic mechanism between dexborneol and pregabalin, exploring both pharmacokinetic and neuropharmacological aspects.

## 2. Material and Methods

### 2.1. Animals

All animal experiments were conducted in accordance with the rules set by the Laboratories Institutional Animal Care and Use Committee (IACUC) of the Chinese Academy of Medical Sciences and Peking Union Medical College (No.00009788). C57BL/6 mice, male, weighing 18–25 g (housed 5 per cage), were obtained from SPF (Beijing) Biotechnology Co., Ltd., Beijing, China. All animals were kept at a constant room temperature of 21–23 °C in a 12:12 h light/dark cycle with ad libitum access to food and water. For behavioral studies, we consistently used 12 animals per group, and for mechanistic studies, the number ranged from 7 to 8 animals per group.

### 2.2. Mouse Model of Spared Nerve Injury and Incisional Pain

**Figure 1:**
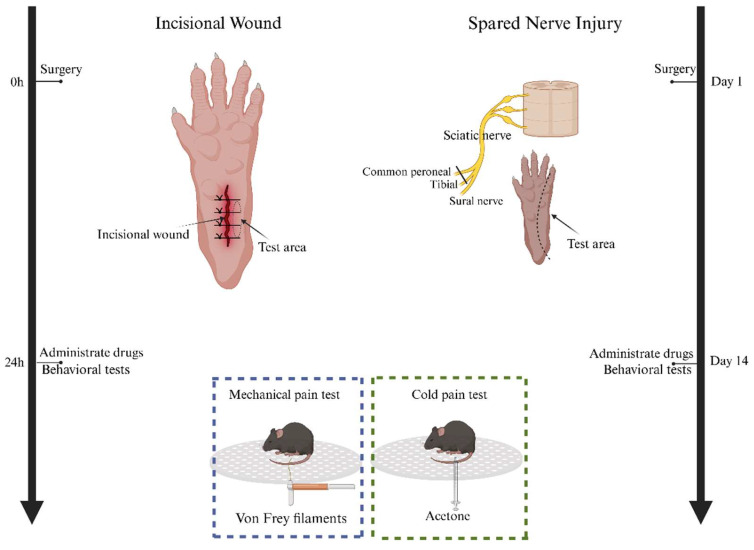
Schematic illustration depicting model operation and study timeline. The timeline begins with the surgical procedures for inducing spared nerve ischemic injury (SNI) and incisional pain (INCI) in rodent models. For SNI, the tibial and common peroneal nerves were ligated, and a segment of the distal nerve stump was removed, while preserving the sural nerve. Behavioral studies for SNI were conducted 14 days post-surgery. For the INCI model, a longitudinal incision was made in the hind paw, accompanied by a simulation of surgical manipulation. Behavioral studies for INCI were conducted 24 h after incision, when pain behaviors reached their peak. The SNI and INCI models were employed to investigate neuropathic and surgical pain, respectively.

The method to induce spared nerve ischemic injury (SNI) in rodents has been previously reported [21] (Figure 1). This procedure involved axotomy and ligation of the tibial and common peroneal nerves, while preserving the sural nerve intact. Specifically, the tibial and common peroneal nerves were tightly ligated, and a 2 mm segment of the distal nerve stump was removed by severing the nerves distal to the ligation site. Care was taken to avoid stretching or contacting the intact sural nerve. The behavioral study was performed 14 days after SNI when animals developed persistent pain behaviors.

We used a mouse model of incisional pain (INCI) that has been previously reported [17] (Figure 1). Briefly, a longitudinal incision was made in the hind paw with a scalpel blade. The blade was initially positioned 2 mm away from the proximal edge of the heel and moved towards the toes, cutting skin and fascia in the movement. The exposed muscle was elevated with tweezers, which were then moved back and forth three times to simulate surgical manipulation; muscle origin and insertion remained intact. The edges of the incision were brought together and gently pressed to allow hemostasis, and the skin was apposed with two single sutures of polypropylene. The behavioral study was performed 24 h after incision, when pain behaviors of the animals reached a peak.

It is important to note that the mice were housed in their regular home cages under standard conditions except during these behavioral experiments.

### 2.3. Behavioral Tests in Mice

To assess mechanical hypersensitivity, mice were housed in plastic cages featuring a metal mesh floor. The mice were housed in their regular home cages except during the behavioral experiments, when they were temporarily placed in plastic cages featuring a metal mesh floor. Following a one-hour habituation period, the plantar surface of the hind paw underwent stimulation using calibrated von Frey hairs (Stoelting, Kiel, WI, USA) of increasing forces (Figure 1). Each filament was applied five times, and the withdrawal threshold was determined when the animal withdrew its paw at least three times. The cut-off value was set at 4 g for mice, and stimuli were administered at a frequency of once per second.

For the evaluation of cold sensitivity, we precisely applied a single drop of acetone to the plantar surface of the hind paw using a 1 mL syringe (Figure 1). This controlled method of acetone delivery enabled us to accurately assess the cold hypersensitivity of the mice, ensuring reproducibility in our measurements. Immediate responses following acetone application were observed and scored for mice as follows: 0 = no response, 1 = startle response without evident paw withdrawal, 2 = withdrawal of the stimulated hind paw, 3 = sustained withdrawal of the stimulated hind paw with flinching or licking.

### 2.4. Pharmacological Treatments

In both the SNI and incisional pain mouse models, the mice were randomly assigned to seven treatment groups: a model group receiving solvent (M), a pregabalin group (P) dosed at 50 mg/kg, a double pregabalin group (2P) dosed at 100 mg/kg, a pregabalin + dexborneol (PB) group receiving 50 mg/kg pregabalin and 300 mg/kg dexborneol, a dexborneol group (B) dosed at 300 mg/kg, a high-dose dexborneol group (B1000) dosed at 1000 mg/kg, and a sham operation group serving as a control. All treatments were administered via gavage at a volume of 0.2 mL per mouse. Each mouse received treatment only one time per experiment. After behavioral tests and treatments, mice were euthanized with isoflurane and decapitated or perfused. The dissection was performed after the animals had undergone a three-hour drug treatment. Following the dissection, the fresh spinal cords were immediately collected and stored at −80 °C to preserve their integrity for further analysis. 

### 2.5. The Calculation Method for Kim Jong-Kyun’s q Value

In this study, Kim Jong-kyun’s q value was employed as a metric to assess whether two distinct drugs exhibit a synergistic inhibitory effect. This calculated value provides a quantitative measure to evaluate drug combinations. The formula for calculating the q value is as follows: q = E_a+b_/(E_a_ + E_b_ − E_a_ × E_b_), where E_a_ and E_b_ represent the inhibition rates of the two inhibitors when acting independently, while E_a+b_ denotes the inhibition rate observed when both inhibitors are combined (i.e., the inhibition rate of the combined medication) [22]. 

The numerator of this formula signifies the “measured combination effect”, whereas the denominator signifies the “expected combined effect”. The interpretation of the q value is based on the following ranges: If (q < 0.85), the effects of the two drugs are antagonistic; if (0.85 ≤ q < 1.15), the effects of the two drugs are simply additive; if (q ≥ 1.15), there is a synergistic effect in the actions of the two drugs [22]. Additionally, to quantify the inhibition of mechanical pain and cold pain, the following formulas were utilized: Inhibition ratio of mechanical pain = (AUC_X_ − AUC_M_)/(32 − AUC_M_) × 100%; inhibition ratio of cold pain = (AUC_M_ − AUC_X_)/(AUC_M_ − 0) × 100%, where AUC_X_ signifies the area under the curve that illustrates the analgesic effects observed in different treatment groups, and AUC_M_ represents the area under the curve that illustrates the pain sensitivity for the model group.

### 2.6. Cells

The BV2 immortalized murine microglial cell line was provided by the Cell Resource Center of Peking Union Medical College (Beijing, China). BV2 cells were constructed by infecting primary microglia with a v-raf/v-myc oncogene-carrying retrovirus (J2). The murine BV2 microglia were cultured in DMEM supplemented with 10% fetal bovine serum (FBS), 100 U/mL penicillin and 100 μg/mL streptomycin, and were maintained in a humidified incubator with 5% CO_2_.

### 2.7. Treatment of BV2 Cells

The BV2 cell suspension was inoculated into a 6-well plate at 10^5^/well, 2 mL per well. After adhesion for about 12 h, the supernatant was discarded, and pregabalin was added to the culture medium containing 10% FBS DMEM to prepare a 10 umol/L pregabalin solution. To prepare a 30 μg/mL solution of dexborneol, a high-glucose medium containing 10% FBS was used. DMSO was added to the medium at a concentration of 0.5% to enhance the solubility of dexborneol. Adding 2 mL to each well (the normal control group use contained 10% fetal bovine serum with DMEM high-glucose culture medium instead), in addition to the blank control group (C), LPS was added at the same time to make the final concentration 200 ng/mL. The in vitro study involved five experimental groups: a blank control group (C), a model group, a pregabalin group (P), a dexborneol group (B), and a combination group of pregabalin and dexborneol (PB). To ensure the reliability of our results, we replicated each of these groups three times. During the study, the cells in these groups were stimulated with LPS for 18 h. Following the stimulation period, the cells were collected for additional analysis to examine the effects of the different treatments.

### 2.8. Real-Time Quantitative PCR Analysis

Total RNA was extracted from cell precipitates and spinal cord tissues using the TRIzol Plus RNA Purification Kit (Invitrogen, Carlsbad, CA, USA). The total RNA concentration was measured using a NanoDrop 2000 spectrophotometer (Thermo Fisher Scientific, Waltham, MA, USA). Purified RNA (1 μg) from each sample was reverse-transcribed into cDNA templates using a HiFi-Script cDNA Synthesis Kit (CWBIO, Taizhou, China). qRT-PCR was performed on an ABI 7500 fast PCR instrument (Applied Biosystems, Foster City, CA, USA) using an UltraSYBR Mixture (Low ROX) (CWBIO, Jiangsu, China) GAPDH was used for normalization. The results were presented as fold changes relative to GAPDH levels and were calculated using the 2^−ΔΔCT^ method.

### 2.9. Detection of Oxidative Stress Levels

We processed cells by centrifuging, discarding the supernatant, dispersing the pellet, and subsequently adding 200 μL of lysis buffer to each well of a 6-well plate to facilitate cell lysis. After lysis, the samples were centrifuged at 12,000× *g* for 5 min at 4 °C, and the resulting supernatant was carefully collected for further analysis. Additionally, spinal cord tissue was precisely weighed, homogenized in normal saline at a 1:9 weight-to-volume ratio, and then centrifuged to extract a 10% homogenate supernatant, also destined for further analysis. Both cell and tissue supernatants were then diluted to various concentrations in preliminary experiments, aiming to determine the optimal concentration for biochemical testing, including assays for superoxide dismutase (SOD), adenosine triphosphate (ATP), reduced glutathione (GSH), and malondialdehyde (MDA), using kits from the Nanjing Jiancheng Bioengineering Institute (Nanjing, China). Protein concentration was accurately determined, and all detections were carried out strictly according to the manufacturer’s instructions.

### 2.10. Pharmacokinetic Experiments with Pregabalin and Dexborneol

Here, 84 male C57 mice were randomly divided into a pregabalin group [50 mg/kg pregabalin, n = 6] and a pregabalin + dexborneol group [50 mg/kg pregabalin + 300 mg/kg dexborneol n = 6]. The two treatment groups were further subdivided based on the time points of microdialysis sampling. Specifically, we had groups for 0.5, 1, 2, 3, 4, 6, and 8 h after the treatment. Each of these subgroups contained 6 animals. After administration, whole blood was collected from mice in each group, and plasma and brain tissue homogenate were prepared. Another 6 mice were used for the collection and preparation of blank plasma and whole brain homogenate.

### 2.11. HPLC-MS/MS 

The sample pretreatment and quantitative analyses were carried out using the method described earlier [23], in which an HPLC-MS/MS system (AB Sciex LLC, Framingham, MA, USA) was applied. Chromatographic conditions: XSelect@ HSS T3 column (2.1 mm × 50 mm, 2.5 μm, Waters Company, Milford, MA, USA); mobile phase is A (water, containing 0.1% formic acid): B (methanol); gradient elution program: 0–1 min, 15% B—60% B; 1.5—3.0 min, 60% B—60% B; 3.0–3.1 min, 60% B—100% B; 3.1–4.5 min, 100% B; 4.50–4.51 min, 100% B—15% B; 4.51–6.0 min, 15% B. The flow rate was 0.3 mL/min, the column temperature was 30 °C, the sample chamber was 4 °C, and the injection volume was 2 μL. Mass spectrometry conditions: a Sciex 6500+ triple quadrupole mass spectrometer equipped with an electrospray ion (ESI) source, curtain gas (N2) 35.0 psi, collision gas (N2) 9 psi, spray voltage 5500 V, atomization gas (N2) 55 psi and auxiliary gas (N2) 55 psi. The turbo-ion spray source temperature was 550 °C. Scans were performed using multiple reaction monitoring (MRM) mode in positive ion mode. The precursors and product ions of pregabalin and gabapentin (internal standard) were 160/142 (Collision energy: 16 V; Declustering potential: 28 V) and 172/154 (Collision energy: 22; Declustering potential: 50 V). Pharmacokinetic parameters were calculated using the noncompartmental model of DAS software 3.0. The maximum plasma concentration (C_max_) and time to peak (T_max_) were both actual measured values, and the area under the time concentration curve (AUC) was calculated using the trapezoidal method.

### 2.12. UPLC-HRMS Analysis for Lipidomic Studies

Untargeted metabolomics analysis utilized a Waters Synapt G2-Si HDMS system paired with an H-class UPLC from Milford, MA, USA. The chromatographic separation employed an Acquity BEH C18 column (2.1 × 50 mm, 1.7 μm). Mobile phase A consisted of a 0.1% formic acid aqueous solution, while mobile phase B comprised acetonitrile with 0.1% formic acid. The gradient elution commenced with 1% mobile phase B for 1.5 min, followed by a linear increase to 100% B over 8.5 min, maintained for 2 min. Injection volume was 2 μL, and column temperature was held at 30 °C. Data acquisition operated in positive mode, scanning within the range of 50–1500 *m*/*z*. The source temperature was set at 150 °C, with a dry gas temperature of 450 °C. MassLynx 4.2 facilitated data acquisition.

### 2.13. Perfusion and Cryo-Sectioning

SNI mice were anesthetized via intraperitoneal injection of 2,2,2-tribromoethanol solution (administered at 250 mg/kg, obtained from Sigma-Aldrich, Darmstadt, Germany). Subsequently, the mice underwent transcardial perfusion with 100 mL of 0.9% saline, immediately followed by perfusion with 250–300 mL of a fixative consisting of 4% paraformaldehyde dissolved in 0.1 M phosphate buffer (PB) at a pH of 7.4. The spinal cord was dissected and post-fixed for 2 h using the same fixative, and then cryoprotected overnight in 25% sucrose. Using a microtome (REM-710, Yamato Koki Industrial, Asaka, Japan), the spinal cord was sliced into 40 μm thick coronal sections. 

### 2.14. Immunofluorescence Staining

The microglia and astrocytes in the spinal cord were detected by immunofluorescence staining using anti-Iba1 antibody and anti-GFAP antibody. First, the sections were incubated in a blocking solution containing 3% normal donkey serum and 0.5% Triton X-100 in 0.1 M PB (pH 7.4) for 1 h. The sections were then transferred to Rabbit anti-Iba1 monoclonal antibody (1:1000; Cat# ab178847; RRID:AB_2832244; Abcam, Hong Kong Administrative Region, Hong Kong, China) and Rat anti-GFAP monoclonal antibody (1:1000; Cat# ab279291; Abcam, Hong Kong Administrative Region, China) in a dilution of 0.1 M PB containing 1% normal donkey serum and 0.5% Triton X-100 for overnight at 4 °C. The following day, after washing three times with 0.1 M PB, the sections were exposed to the secondary antibodies of Alexa Fluor (AF) 594 donkey anti-rabbit IgG (1:500; Cat# A21207; RRID: AB_141637; Thermo Fisher, Waltham, MA, USA), AF 488 donkey anti-rat IgG (1:500; Cat# A21208; RRID: AB_2535794; Thermo Fisher, Waltham, MA, USA), and 4′,6-diamidino-2-phenylindole (1:50,000; Cat# D3570; Thermo Fisher, Waltham, MA, USA) for 2 h at room temperature, then washed with 0.1 M PB. The sections were then mounted on the microscope slides and coverslipped with 50% glycerin. The samples were viewed using the Virtual Slide System (VS120, Olympus, Tokyo, Japan), and representative regions were selected to view in more detail using a confocal imaging system (FV1200, Olympus).

### 2.15. Chemicals

Dexborneol was obtained from Chengdu DeSiTe Biological Technology Co., Ltd. (Chengdu, China). Pregabalin was supplied by Shanghai Aladdin Biochemical Technology Co., LTD. (Shanghai, China). MS-grade methanol, acetonitrile, water, and formic acid were purchased from Fisher Scientific (Waltham, MA, USA). Deuterated lipid mediators (LMs) were purchased from Cayman (Ann Arbor, MI, USA). All other reagents were of analytical or HPLC grade.

## 3. Statistics

All data were analyzed using GraphPad Prism 9.0 (GraphPad Software, San Diego, CA, USA) and are presented as means ± standard error of the mean (SEM). Unpaired *t*-tests were used for comparisons between two groups of continuous variables. One-way analysis of variance (ANOVA) followed by a post hoc test was employed for comparisons among three or more groups. The Kolmogorov–Smirnov test was employed to confirm the normality of the dataset. For the comparison of values at distinct time points relative to baseline measurements, either a two-way ANOVA with repeated measures followed by Dunnett’s multiple comparisons test or the Wilcoxon signed-rank test was conducted. To evaluate the effects of the drug group versus the model group at specific time points, we used either a two-way ANOVA with repeated measures followed by Bonferroni’s multiple comparisons test. Additionally, data represented by the area under the curve underwent a one-way ANOVA, with further inter-group significance testing performed using Tukey’s multiple comparisons test. Significance was assessed by comparing the values for independent groups as follows: * *p <* 0.05; ** *p <* 0.01; *** *p <* 0.001 or # *p <* 0.05; ## *p <* 0.01; ### *p <* 0.001. Results with *p <* 0.05 were considered statistically significant.

## 4. Results

### 4.1. Dexborneol Augments Pregabalin’s Analgesic Effect in a Mouse Model of Peripheral Nerve Injury

In this study, we explored whether dexborneol can boost pregabalin’s analgesic potency, overcoming its therapeutic limitations, through experiments with animal models of peripheral neuropathic pain (induced by SNI) and incisional pain. In general, our study revealed that the analgesic potency of pregabalin combined with dexborneol was significantly superior to pregabalin alone.

**Figure 2:**
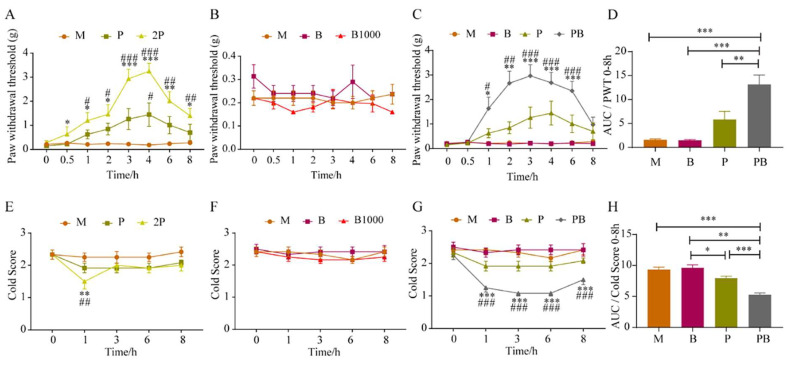
The synergistic analgesic effects of dexborneol and pregabalin in mice with SNI-induced peripheral neuropathic pain. Data represent mean ± SEM. n = 12 animals/group. The six treatment groups are model (M; solvent), pregabalin (P; 50 mg/kg), double pregabalin (2P; 100 mg/kg), pregabalin + dexborneol (PB; 50 mg/kg pregabalin + 300 mg/kg dexborneol), dexborneol (B; 300 mg/kg), and high-dose dexborneol (B1000; 1000 mg/kg). (**A**,**E**) exhibit pregabalin’s effects on mechanical and cold hypersensitivities. (**B**,**F**) show dexborneol’s impact on mechanical and cold hypersensitivities. (**C**,**G**) illustrate the combined effects of pregabalin and dexborneol. (**D**,**H**) present the AUC analysis for mechanical and cold hypersensitivities for different treatment groups. Statistical significance is denoted by * *p* < 0.05, ** *p* < 0.01, *** *p* < 0.001 when compared to baseline or using AUC, and # *p* < 0.05, ## *p* < 0.01, ### *p* < 0.001 when compared to the model group.

Initially, we examined the analgesic impact of pregabalin at two distinct doses on SNI mice. Referring to (Figure 2A), when compared to its baseline, the paw withdrawal threshold (PWT) in the 2P group exhibited a notable increase from 0.5 h onward, persisting until 8 h. However, in the P group, pregabalin had a minimal effect on the mechanical pain threshold of SNI model mice, showing a significant difference from the Model group only at the 4 h timepoint. Moreover, our results demonstrated that dexborneol, both B and B1000 treatments, did not exhibit significant analgesic effects on mechanical pain in SNI mice (Figure 2B). Additionally, no alterations were observed in the mice’s normal physiological parameters, including respiration and heart rate, indicating the safety of these dexborneol doses.

Nonetheless, we discovered that the simultaneous administration of pregabalin and dexborneol at low doses could significantly mitigate the mechanical pain sensitivity exhibited by SNI mice. As clearly demonstrated in Figure 2C, a comparative analysis encompassing the P group, B group, and PB group revealed that the mechanical pain threshold in SNI model mice from the PB group was markedly higher compared to both the P and B groups at 1, 2, 3, 4, and 6 h following drug administration. The area under the curve (AUC) was employed to evaluate and compare the overall intensity of mechanical pain across all SNI groups within the 0–8 h timeframe. Notably, the PB group also demonstrated a significantly superior analgesic effect compared to the P group, B group, and the Model group (Figure 2D).

Analogous to observations in mechanical pain scenarios, our findings revealed that the 2P group demonstrated a significant analgesic effect on cold pain (Figure 2E) in SNI mice. Specifically, at the 1 h post-administration time point, the cold score in the 2P group was significantly decreased in comparison to the M group. In addition, the P group did not exhibit a considerable impact on the cold pain threshold. Furthermore, as depicted in Figure 2F, the PB treatment did not evoke a discernible analgesic effect on cold pain in SNI mice. 

A comprehensive comparison between the P group, B group, and PB group clearly indicated that, as shown in Figure 2G, only the PB group demonstrated a significant decrease in cold score at 1, 3, 6, and 8 h after drug administration. Both the P and the B groups failed to show statistically meaningful differences from the M group. To assess the overall analgesic effectiveness of the various groups over the 0–8 h period, we computed the AUC. Notably, the analgesic effect observed in the PB group was significantly superior to that in the P group, B group, and M group (Figure 2H). 

By calculating the AUC of each group, the inhibition rates for mechanical pain and cold pain in the P group, B group and PB group were calculated, respectively, taking the M group and the ideal analgesic state as reference. The calculation showed that Kim Jong-kyun’s q value for mechanical pain and cold pain in the PB group in the SNI model was 2.79 and 3.59, respectively, and the two drugs showed obvious synergistic effects on mechanical pain and cold pain (Table 1).

### 4.2. Dexborneol Amplifies Pregabalin’s Analgesic Effectiveness in a Mouse Model of Incisional Pain

**Figure 3:**
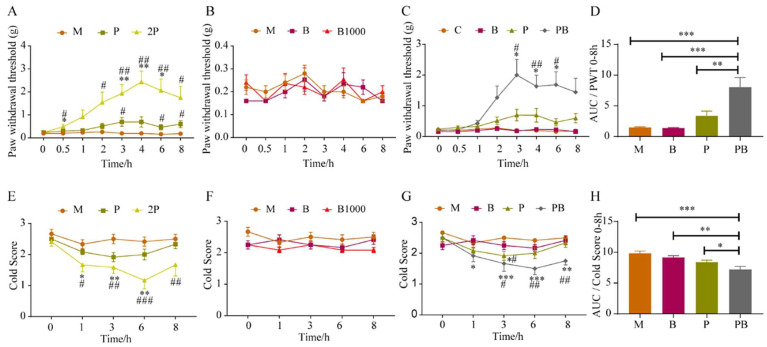
The analgesic effects of pregabalin and dexborneol, alone and in combination, on mice with incision-induced post-operative pain. Data are represented as mean ± SEM (n = 12 animals/group). There were six treatment groups: model (M; administered solvent only), pregabalin (P; 50 mg/kg), double pregabalin (2P; 100 mg/kg), pregabalin + dexborneol (PB; 50 mg/kg pregabalin combined with 300 mg/kg dexborneol), dexborneol (B; 300 mg/kg), and high-dose dexborneol (B1000; 1000 mg/kg). (**A**,**E**) exhibit the effects of pregabalin at two doses on mechanical and cold hypersensitivities. (**B**,**F**) demonstrate the impact of dexborneol at two doses on the same measures. (**C**,**G**) show the combined analgesic effects of pregabalin and dexborneol. (**D**,**H**) display the results of AUC analysis for mechanical and cold hypersensitivities of different groups. Statistical significance is indicated by * *p* < 0.05, ** *p* < 0.01, *** *p* < 0.001 when compared to the baseline or using AUC, and # *p* < 0.05, ## *p* < 0.01, ### *p* < 0.001 when compared to the model group.

To further validate the analgesic spectrum of this drug combination, we adopted a mouse model of incisional pain. Figure 3A illustrates that pregabalin yielded marked analgesic effects in mice with incisional pain, effectively reducing mechanical hypersensitivity as assessed by von Frey hair testing. 2P treatment significantly ameliorated mechanical pain when compared to its baseline or the M group, with analgesic effects persisting from approximately 30 min to 8 h. A lower dose in the P group showed a lesser influence on the mechanical pain threshold in these mice, with statistically significant deviations from the M group observed only at the 3, 6, and 8 h timepoints. In addition, our observations indicated that the B and B1000 groups failed to elicit significant analgesic effects on mechanical hypersensitivity when tested using von Frey hairs (Figure 3B). Moreover, our data revealed no discernible alterations in the mice’s baseline physiological parameters, including respiration and heart rate. 

Figure 3C presents a comprehensive comparison between the M group, P group, B group, and PB group, clearly indicating that only the PB group showed a significantly elevated mechanical pain threshold in SNI mice in comparison to the M group at the 3, 4, and 6 h time intervals. With reference to Figure 3D, AUC was employed to evaluate and contrast the overall analgesic efficacy of different groups on mechanical pain within the 0–8 h time interval (following treatment). Apparently, the analgesic effect exhibited by the PB group was distinctly better than that of the P group, B group, or M group.

As illustrated in Figure 3E, it is apparent that 2P group experienced significant analgesic effects against cold pain within the 1–8 h time interval in the incision pain model. Nevertheless, pregabalin at a reduced dose did not have a substantial effect on the cold pain threshold in the P group. Moreover, Figure 3F indicates that dexborneol in the B and B1000 groups failed to demonstrate discernible analgesic effects on cold pain. 

The extensive evaluation encompassing the M group, P group, B group, and PB group unveiled that only the PB group experienced a substantial decrease in cold pain scores among mice subjected to the incisional pain at the 3, 6, and 8 h timepoints (Figure 3G). By comparison, the P group displayed a minor yet notable reduction in the cold pain score at the 3 h timepoint relative to the M group, whereas the B group did not exhibit any significant analgesic effects (Figure 3G). With reference to Figure 3H, AUC was utilized to gauge the cumulative analgesic potency on cold pain in the different groups. Clearly, the analgesic impact observed in the PB group surpassed that of the P group, B group, and the M group in a statistically significant manner.

By calculating the AUC of each group, the mechanical pain and cold pain inhibition rates in the of P group, B group and PB group were calculated, respectively, taking the M group and the ideal analgesic state as reference. The calculations showed that that Kim Jong-kyun’s q value for PB group mechanical pain and cold pain in the incisional pain model was 3.54 and 1.32, respectively, and the two drugs showed an obvious synergistic effect on mechanical pain and cold pain (Table 1).

### 4.3. Dexborneol Can Promote Pregabalin Entry into the Central Nervous System to Exert Analgesic Effect

In the pharmacokinetics study, we examined dexborneol’s effect on pregabalin’s absorption into the blood stream upon oral administration and its penetration into the CNS through the BBB, with experimental results demonstrating that dexborneol promotes pregabalin’s entry into the blood circulation and CNS, thereby enhancing its analgesic effect.

Specifically, after intragastric administration, the pregabalin concentration in the CNS exhibited a distinct trend compared to that in plasma; the T_max_ value of pregabalin, whether administered alone or in combination with dexborneol, was higher in CNS tissue compared to plasma (Table 2).

**Figure 4:**
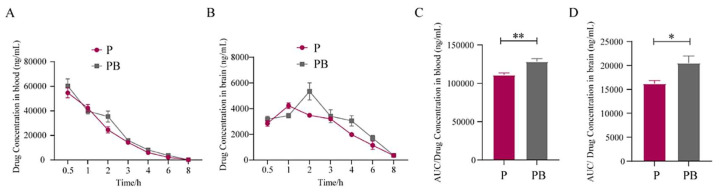
The pharmacokinetic analysis shows dexborneol’s effect on pregabalin absorption and BBB penetration. Data are presented as mean ± SEM, n = 6 animals/group. (**A**) Blood pregabalin levels after oral dosing with pregabalin alone (50 mg/kg, P group) vs. pregabalin 50 mg/kg + dexborneol 300 mg/kg (PB group), over 8 h. (**B**) Brain pregabalin levels of the two treatment groups at different time points. (**C**,**D**) AUC analysis for pregabalin blood and brain levels, highlighting the differences between the two treatment groups. Statistical significance is denoted by * *p* < 0.05 and ** *p* < 0.01.

When pregabalin was administered as a monotherapy (P group), it entered the bloodstream swiftly, reaching peak concentrations at 0.5 h post-administration. Pregabalin could also be detected in the CNS at this time point, although its peak brain concentration was observed at 1 h, slightly lagging behind its blood peak (Figure 4A,B; Table 2). The overall cerebral blood penetration ratio for the P group was approximately 14.62%. 

When pregabalin was administered in combination with dexborneol (PB group), its blood concentration peaked at 0.83 h, indicating a slight delay and a marginal increase compared to monotherapy. However, a significant difference was evident in the CNS, where pregabalin reached its peak concentration at 2 h, one hour later than when administered alone (Figure 4A,B; Table 2). As a result, the brain blood penetration ratio rose to 16.00%, representing a modest enhancement compared to the P group.

The elevation of pregabalin levels facilitated by dexborneol was further corroborated by an increase in C_max_ values observed in both plasma and the CNS when dexborneol was administered concomitantly. Moreover, the AUCs of the PB group were significantly higher than those of the P group in both plasma and CNS (Figure 4C,D), indicating a notably greater overall drug concentration within the 8 h observation period. These findings confirmed that dexborneol facilitates the absorption of pregabalin and enhances its penetration through the BBB into the CNS.

### 4.4. Pregabalin-Dexborneol Suppresses Glial Cell Activation in a Mouse Model of Peripheral Nerve Injury

Microglia, which resemble macrophages in the CNS, become activated following nerve damage. This activation triggers the release of inflammatory mediators, amplifying glutamatergic excitatory synapse transmission and the conveyance of pain signals to the brain [24]. We investigated whether PB treatment can modulate this process by employing SNI animal models and BV2 microglial cells stimulated with lipopolysaccharide (LPS).

**Figure 5:**
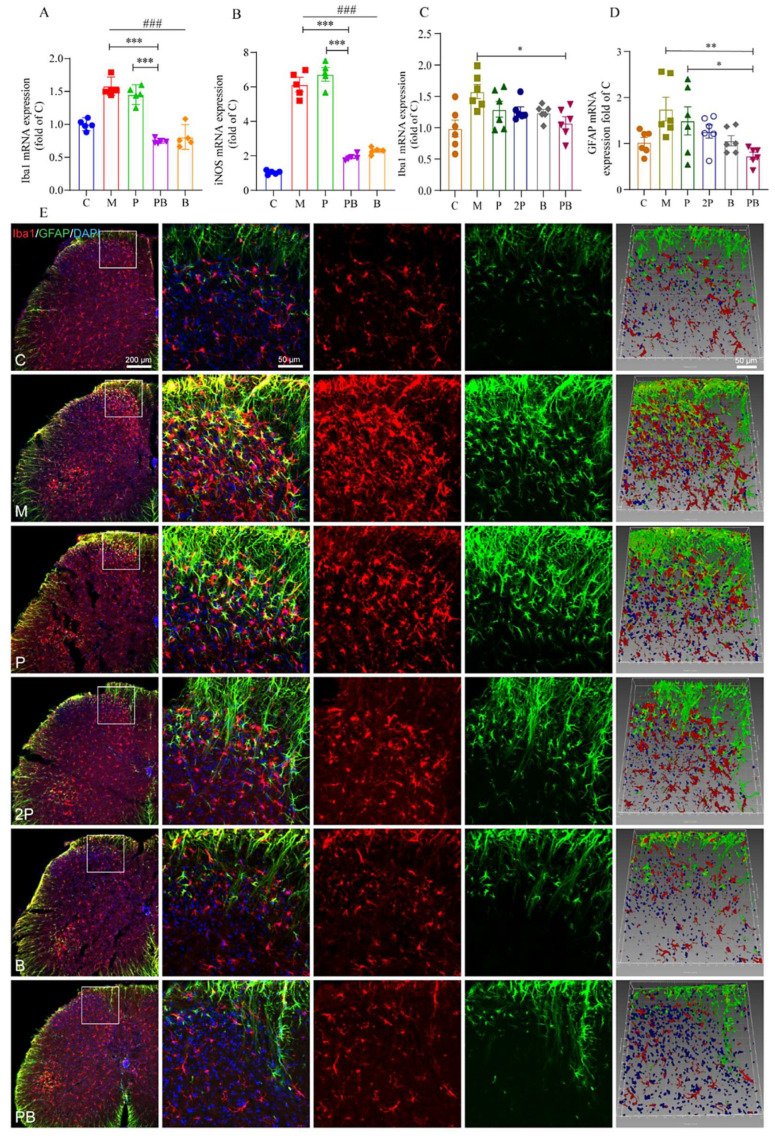
The impact of pregabalin and dexborneol combination on glial cell activation in the spinal cord dorsal horn of mice with SNI. Data are presented as mean ± SEM. (**A**,**B**) The gene expression levels of Iba1 and iNOS in BV2 cells, determined using qRT-PCR (n = 5 animals/group). (**C**,**D**) The gene expression of Iba1 and GFAP in spinal cord tissue from SNI and control mice, also measured via qRT-PCR (n = 6 animals/group). Statistical significance is denoted by * *p* < 0.05, ** *p* < 0.01, *** *p* < 0.001 and ### *p* < 0.001. (**E**) Representative images of immunofluorescent staining for Iba1 (red), GFAP (green) and DAPI (blue) in the spinal cord (scale bar, 200 μm). Higher magnification of the boxed regions showing the labeled microglia and astrocytes in more detail (scale bar, 50 μm). Adjusted images from higher magnification with three-dimensional reconstruction in a sloping pattern (scale bar, 50 μm).

As shown in Figure 5A, when BV2 cells were stimulated with LPS, the expression of Iba1 mRNA increased significantly, peaking after 24 h of LPS treatment. However, the combined administration of pregabalin and dexborneol effectively suppressed this activation in vitro. In contrast, pregabalin alone did not demonstrate a reduction in Iba1 expression. This suggests that PB treatment inhibits LPS-induced BV2 cell activation in a laboratory setting.

Inducible nitric oxide synthase (iNOS) expression serves as an additional indicator of microglial activation. As depicted in Figure 5B, there was a notable decrease in iNOS mRNA levels in both the PB and B groups when compared to the M group. Nevertheless, no discernible difference was observed between the P group and the M group. Hence, it is worth emphasizing that pregabalin on its own did not exert any inhibitory effect on the activation of BV2 cells. 

Similarly, in the spinal cord of SNI mice, the activation of microglial cells was suppressed. Specifically, the expression of Iba1 was significantly reduced in the PB group compared to the M group (Figure 5C). Additionally, there was no significant difference detected in the levels of Iba1 mRNA between the P and 2P groups. However, in contrast to both the P and 2P groups, the PB group demonstrated a reduced expression of Iba1 mRNA in the spinal cords of SNI mice. The immunofluorescence results further supported these in vivo findings. In the C group, the resident microglial cells were in “resting” state with small cell bodies and fine processes. In contrast, in the M group, numerous activated microglial cells gathered within injured territories and displayed shortened processes and enlarged somata. Despite the absence of marked alterations in the P and 2P groups compared to the M group, the B and PB groups exhibited a trend of decreased immunofluorescence of Iba1, indicating a reduction in activated microglia (Figure 5E).

Our research indicates that glial fibrillary acidic protein (GFAP), a crucial biomarker for astrocyte activation, showed significantly elevated expression levels in the spinal cord tissue of mice with SNI. Albeit compared to the M group, the PB group exhibited a significant decrease in GFAP expression (Figure 5D), suggesting that the combined treatment led to a substantial reduction in astrocyte activation. Interestingly, neither the P nor the 2P group showed any suppression of GFAP expression, indicating pregabalin alone was insufficient to reduce central astrocyte activation in the SNI model (Figure 5D). These findings were further supported by immunofluorescence results, which align with our in vivo observations. Notably, despite the absence of perceptible differences between the P and 2P groups and the M group, the B and PB groups demonstrated a discernible decrease in the immunofluorescence that characterizes GFAP+ astrocytes (Figure 5E).

### 4.5. Pregabalin-Dexborneol Suppresses Detrimental SNI-Induced Neuroimmune Crosstalk and Neuroinflammation via HMGB1/TLR4/NF-κB Inhibition

**Figure 6:**
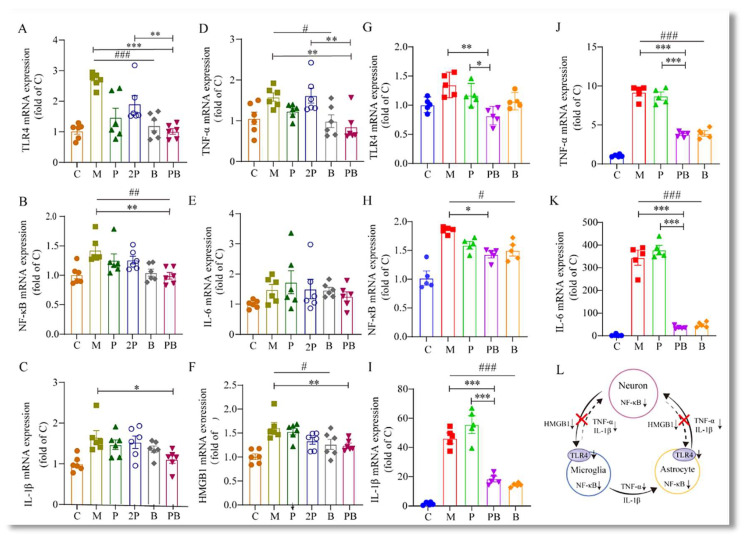
Pregabalin-dexborneol disrupts the HMGB1-fueled inflammatory cycle. Data are presented as mean ± SEM. (**A**–**F**) The mRNA expression levels of TLR4, NF-κB, TNF-α, IL-6, IL-1β, and HMGB1 in spinal cord tissue of SNI or control mice, assayed by qRT-PCR (n = 6 animals/group). (**G**–**K**) The mRNA expression of TLR4, NF-κB, TNF-α, IL-6, and IL-1β in BV2 cells, also determined via qRT-PCR (n = 5 animals/group). Statistical significance is marked by * *p* < 0.05, ** *p* < 0.01, and *** *p* < 0.001; # *p* < 0.05, ## *p* < 0.01, and ### *p* < 0.001. (**L**) A schematic diagram illustrating how pregabalin-dexborneol hinders harmful neuroimmune crosstalk and neuroinflammation induced by SNI through the inhibition of the HMGB1/TLR4/NF-κB pathway.

Neurons, upon external stimulation, secrete HMGB1, which binds to TLR4 receptors on glial cells, initiating a cascade of reactions. We found that in the spinal cord of mice subjected to SNI, the expression of HMGB1 mRNA was significantly upregulated compared to the naïve control group (Figure 6F). 

Interestingly, when pregabalin and dexborneol were administered together, we observed a significant reduction in the expression of HMGB1, TLR4, and NF-κB mRNA, compared to the M group (Figure 6A,B,F). This finding indicates that the combined administration of pregabalin and dexborneol effectively suppresses the neuroimmune interaction triggered by HMGB1 secretion. However, it is worth noting that this suppressive effect was not observed in the P group, suggesting a synergistic action between pregabalin and dexborneol in modulating this neuroimmune response.

In our in vitro experiments, as shown in Figure 6G–K, LPS stimulation resulted in notable alterations in the production of inflammatory mediators, specifically including TLR4, NF-κB, TNF-α, IL-6, and IL-1β. We confirmed that the PB treatment significantly suppressed the expression of TLR4 and NF-κB in BV2 cells (Figure 6G,H). Specifically, upon LPS stimulation, we observed a notable increase in the mRNA levels of TLR4 and NF-κB genes in BV2 cells. However, the PB treatment combination markedly attenuated these LPS-induced changes. These findings implicate that PB treatment not only prevents the activation of BV2 cells but also blocks the production of inflammatory mediators via the TLR4-NF-κB signaling pathway.

Moreover, the concomitant administration of pregabalin and dexborneol significantly decreased the mRNA expression of inflammatory factors, such as IL-1β, IL-6, and TNF-α, secreted by glial cells. This observation was evident in both in vivo and in vitro experiments (Figure 6C–E,I–K), again confirming that the PB combination potentially disrupts the production of inflammatory mediators through the TLR4-NF-κB signaling pathway.

These findings reveal that pregabalin-dexborneol mitigates SNI-induced harmful neuroimmune interactions and neuroinflammation through HMGB1/TLR4/NF-κB suppression (Figure 6L), emphasizing the potential of PB therapy in treating neuropathologies characterized by microglial activation and inflammation.

### 4.6. Pregabalin-Dexborneol Activates Nrf2 to Regulate Oxidative Stress Levels and Disrupts a Self-Amplifying Oxidative Cycle in a Mouse Model of Peripheral Nerve Injury

Oxidative stress serves as a catalyst for neuropathy and inflammation, leading to the onset and continuation of pain. It also causes neuronal DNA damage and disrupts multiple signaling pathways, ultimately leading to demyelination and hypersensitivity of signal transmission [25]. Oxidative stress pathways are closely connected to various signal transduction cascades and transcription factors, including NF-κB, COX-2, Nrf2, and inflammatory cytokines like TNF and IL-1β [26]. Additionally, there exists a positive feedback loop between inflammation and oxidative stress, where increased oxidative stress drives further inflammation, and inflammation, in turn, fuels more oxidative stress. Both processes involve the same key factors and perpetuate each other. Superoxide dismutase (SOD) and malondialdehyde (MDA) are key indicators for assessing oxidative stress levels, where SOD scavenges superoxide anion free radicals to protect cells, while MDA represents the degree of lipid peroxidation due to reactions with oxygen free radicals [27].

**Figure 7:**
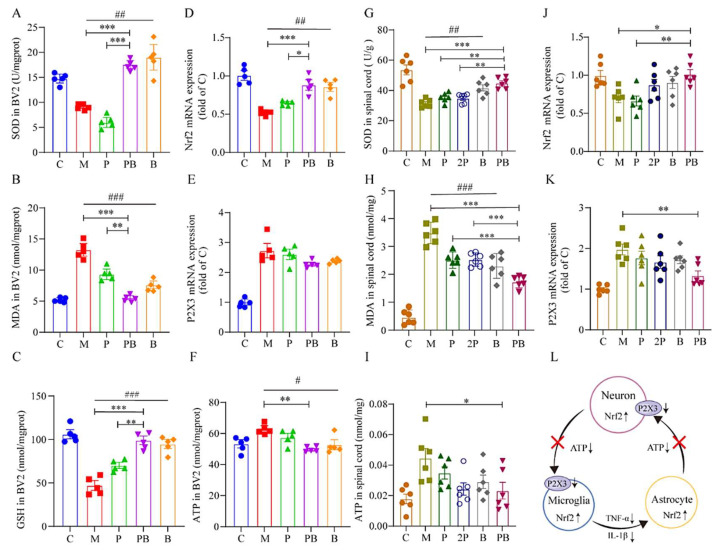
Nrf2 activation induced by pregabalin-dexborneol disrupts the self-perpetuating oxidative cycle. Data are presented as mean ± SEM. (**A**–**C**) SOD, MDA, and GSH activity in BV2 cells. (**D**,**E**) The mRNA expression levels of Nrf2 and P2X3 in BV2 cells, determined using qRT-PCR (n = 5 animals/group). (**F**) ATP activity in BV2 cells (n = 5 animals/group). (**G**–**I**) SOD, MDA, and ATP activity in spinal cord tissue of SNI or control mice (n = 6 animals/group). (**J**,**K**) The mRNA expression of Nrf2 and P2X3 in spinal cord tissue of SNI or control mice, also assayed by qRT-PCR (n = 6 animals/group). Statistical significance is denoted by * *p* < 0.05, ** *p* < 0.01, and *** *p* < 0.001; # *p* < 0.05, ## *p* < 0.01, and ### *p* < 0.001. (**L**) A schematic diagram outlining how pregabalin-dexborneol activates Nrf2 to modulate oxidative stress levels, thereby disrupting the self-amplifying oxidative cycle.

Our testing indicated a substantial decrease in SOD enzyme activity and an increase in MDA content in both LPS-stimulated BV2 cells and the spinal cords of SNI model mice (Figure 7A,B,G,H). We discovered that oxidative stress was notably more effectively mitigated in the PB group than in the P group. Specifically, following LPS stimulation in vitro, MDA levels in BV2 cells escalated remarkably. However, upon drug administration, MDA content decreased in groups P, B, and PB, with the PB group exhibiting the most significant decline (Figure 7B). The Nrf2 and SOD results revealed that only the B and PB groups demonstrated a notable increase compared to the M group, suggesting that dexborneol primarily contributes to the in vitro antioxidative activity (Figure 7A).

As depicted in Figure 7G, the SOD activity in the spinal cords of SNI model mice was significantly lower than in naïve mice (C group). When compared to the M group, the P or 2P group’s SOD activity level remained unaffected. However, the SOD activity in the PB group showed a considerable increase, exhibiting significant differences when compared to both the M group and the P group. In the assessment of MDA levels (Figure 7H), a substantial increase in MDA content was observed in the spinal cord of SNI model mice compared to naïve mice. When compared to the M group, MDA levels decreased in all other groups, with the most significant reduction occurring in the PB group. Significant differences were evident when contrasting the PB group with the P and 2P groups. Additionally, Nrf2 results indicated that only the B and PB groups showed a marked increase in comparison to the M group, confirming that dexborneol is the primary contributor to the in vivo antioxidative activity (Figure 7J).

The P2X3 receptor, an ATP-gated non-selective cation channel of the P2X family, is abundantly present in the nervous system. Upon activation by ATP, it initiates a non-selective cation influx, cell depolarization, and subsequent activation of related receptors, thereby mediating signals crucial for regulating neuronal excitability [28]. In our study, we observed that under in vitro conditions, LPS stimulation increased ATP content in BV2 cells (Figure 7F), while only the pregabalin-dexborneol combination group (PB) effectively reduced ATP production in BV2 cells (Figure 7F). However, across all treatment groups, there was no significant decrease in P2X3 receptor expression (Figure 7E). This could be explained by the fact that P2X3 receptors are primarily located on nerve cell membrane [29]. In in vivo experiments, we observed a significant increase in ATP content in the spinal cord tissue of SNI mice, which subsequently decreased after drug administration (Figure 6I). Although there was a trend towards reduction in the P, 2P, B, and PB treatment groups, only the PB group demonstrated a statistically significant effect, with results comparable to the C group (Figure 7I). Similarly, for P2X3 expression, only the PB group revealed significantly reduced P2X3 levels in the spinal cord tissue of SNI mice (Figure 7K).

Our study demonstrates that dexborneol regulates oxidative stress via Nrf2 gene activation, reducing ATP release and pain transmission, while the combined treatment with pregabalin inhibits microglia activation in the CNS, further reducing ATP production and neuronal depolarization (Figure 7L).

### 4.7. Dexborneol Exerts Analgesic Modulatory Effects through Lipid Mediators in a Mouse Model of Peripheral Nerve Injury

**Figure 8:**
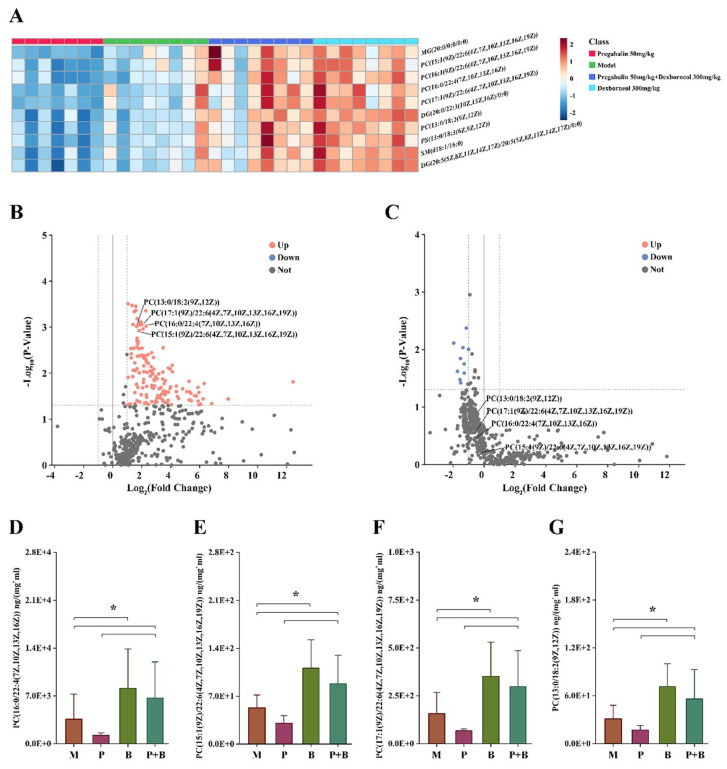
Lipid metabolism regulation induced by pregabalin-dexborneol intervention. Data are presented as mean ± SEM (n = 7–8 animals/group). (**A**) Heatmap analysis of the top differentiated lipids. (**B**,**C**) Volcano analysis of lipids in serum (pregabalin 50 mg/kg + dexborneol 300 mg/kg vs. pregabalin 50 mg/kg, pregabalin 50 mg/kg + dexborneol 300 mg/kg vs. dexborneol 300 mg/kg). (**D**–**G**) Differential PCs (16:0/22:4(7Z,10Z,13Z,16Z)), PC (15:1(9Z)/22:6(4Z,7Z,10Z,13Z,16Z,19Z)), PC (17:1(9Z)/22:6(4Z,7Z,10Z,13Z,16Z,19Z)), PC (13:0/18:2(9Z,12Z)) levels in serum between groups. * *p* ≤ 0.05.

In this study, we employed state-of-the-art lipidomics technology to investigate the role of lipids in mediating the analgesic activity of PB treatment in SNI mice. A total of 480 lipid species were identified, comprising 113 PCs, 20 PEs, 36 PS, 36 PGs, 28 PIs, 24 Pas, 49 DGs, 84 TGs, 20 SMs, and 70 Cers. One-way ANOVA analysis was conducted to discern notable differences between PB and P. Of these lipids, 101 species exhibited a p-value of less than 0.05. PCs emerged as the most significantly altered lipid group, constituting approximately 50% (42 out of 101) of the altered lipids. Through volcano analysis comparing PB and P, lipids demonstrating both significance (*p* < 0.05) and a substantial fold change (FC > 1.5) were cataloged (refer to Table 3). Among these, phosphatidylcholines (PCs) containing unsaturated fatty acids, particularly those with polyunsaturated fatty acids (DHA, ADA, LA), showed the most pronounced changes (Figure 8). All four metabolites depicted in the figure are constituents of PC, the most prevalent phospholipid found in mammalian cell membranes. PC plays a crucial role in regulating lipid, lipoprotein, and systemic energy metabolism [30]. Among these metabolites, PC (15:1(9Z)/22:6 (4Z,7Z,10Z,13Z,16Z,19Z)) and PC (17:1(9Z)/22:6 (4Z,7Z,10Z,13Z,16Z,19Z)) contain docosahexaenoic acid (DHA). Research has demonstrated DHA’s potent antioxidant properties, which can influence cellular physiological activities by regulating mitochondrial function. DHA effectively scavenges reactive oxygen species generated during cellular aerobic respiration, thereby restoring antioxidant homeostasis [31]. Moreover, it enhances the activity of mitochondrial dehydrogenases, mitigating the adverse effects of oxidative free radicals on mitochondria [32]. Furthermore, DHA exhibits antioxidant effects by modulating various signaling pathways. It induces the activity of anti-inflammatory mediators and suppresses the expression of pro-inflammatory genes, thereby bolstering the survival of oxidatively stressed cells. These effects are mediated through pathways such as PI3K/Akt and mTOR/p70S6K, ultimately enhancing cellular antioxidant properties [33,34].

## 5. Discussion

The anticonvulsant pregabalin, widely prescribed as a first-line therapy for chronic pain, may not always demonstrate optimal effectiveness in specific clinical situations. This frequently necessitates higher doses, which unfortunately often lead to significant side effects [35]. Our research demonstrates that dexborneol alone does not have analgesic properties in SNI and incisional pain models. Yet, when combined with pregabalin, dexborneol increases pregabalin’s analgesic potency and extends the duration of pain relief. Given pregabalin’s limitations and side effects, our finding that dexborneol boosts pregabalin’s analgesic effect bears substantial clinical importance. This unique therapeutic approach could potentially reduce pregabalin’s associated side effects and improve its overall therapeutic efficacy, presenting a new strategy to refine pain management. The specific advantages of this combination and its mechanistic basis are elaborated in the subsequent sections.

### 5.1. Dexborneol Enhances Pregabalin’s Ability to Enter the CNS

**Figure 9:**
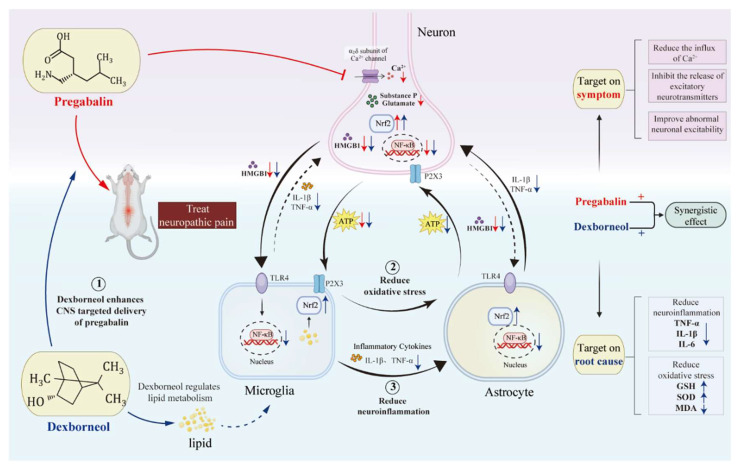
Dexborneol enhances pregabalin’s analgesic effect by suppressing CNS microglial activation via antioxidant and anti-inflammatory pathways. The red arrows and lines indicate the direction and magnitude of the effect of pregabalin, while the blue arrows and lines represent the corresponding effects of dexborneol. Pregabalin binds to the α2δ1 subunit, decreasing glutamate receptor (AMPA, NMDA) expression and phosphorylation. This downregulation blocks glutamate neurotransmission and also enhances GABA neurotransmission, reducing neuronal excitability and contributing to analgesia. Dexborneol facilitates pregabalin’s CNS penetration, disrupting self-amplifying inflammatory and oxidative cycles by inhibiting the TLR4/NF-κB pathway, upregulating Nrf2, and exerting anti-oxidative effects through lipid modulation. This synergistic approach targets both symptoms and root causes of neuroinflammation, providing profound analgesic effects.

As the main constituent of natural borneol in traditional Chinese medicine, dexborneol plays a crucial role in directing medicines to the central nervous system (CNS). Numerous pharmacological studies have validated its efficiency in aiding drug absorption, enhancing blood–brain barrier (BBB) permeability, and extending drug retention time, all without causing toxic side effects [36]. In accordance, our pharmacokinetic studies show dexborneol helps pregabalin to reach the circulatory system and CNS tissue, thereby boosting its analgesic potency (Figure 9). 

We predict that dexborneol improves the delivery efficiency of pregabalin to the CNS through multiple synergistic mechanisms. This is primarily achieved through several means: Firstly, dexborneol may act as a substrate or inhibitor binding to efflux pumps such as P-glycoprotein transport sites [37], thereby competitively inhibiting the pumping out of pregabalin and allowing it to accumulate to higher concentrations within the cell. Secondly, dexborneol can also alter the permeability of cell membranes, possibly through interactions with lipids in the biological membrane [38], thereby enhancing pregabalin’s ability to penetrate cell membranes. Lastly, dexborneol may further facilitate pregabalin’s transmembrane transport by enhancing the expression or activity of specific transporters such as LTA1 (L-type Amino Acid Transporter 1), which is considered to be the active transporter of pregabalin [39]. Although these mechanisms indicate the potential of dexborneol in improving drug delivery, further validation is still necessary to confirm the precise way dexborneol enhances the central delivery of pregabalin.

### 5.2. Pregabalin Modulates Neuronal Signal Transmission 

Dexborneol not only facilitates pregabalin’s entry into the CNS but also contributes to a broader synergistic effect. This combination modifies neuronal signal transmission and changes the CNS microenvironment, effectively overcoming barriers that usually impede recovery from neuronal abnormalities. 

When nerve damage occurs, neurons within the pain-relaying network exhibit an increased reactivity, tending to depolarize more easily. This enhanced excitability leads to persistent pain sensations. The altered neuronal excitability and subsequent pain signaling are key factors in the pathophysiology of chronic pain [40].

Pregabalin’s primary effects are centered on neural modulation to reduce pain signals. Its key therapeutic mechanism involves interacting with the α2δ1 calcium channel subunit in neurons, which is essential for pregabalin’s capacity to adjust pain signaling and offer relief from neuropathic and other types of pain conditions [41].

Specifically, pregabalin binding to the α2δ1 subunit reduces the expression and phosphorylation of glutamate receptors, particularly the N-Methyl-D-aspartic acid (NMDA) and α-amino-3-hydroxy-5-methyl-4-isoxazolepropionic acid (AMPA) receptors. By downregulating the activity of these receptors, pregabalin blocks glutamate neurotransmission, which is a key process in the generation and propagation of pain signals [42]. Moreover, pregabalin mimics the activity of GABA in the central nervous system, enhancing GABA neurotransmission, which leads to a reduction in neuronal excitability. This mechanism further contributes to pregabalin’s analgesic effects [43].

### 5.3. Dexborneol Alters the CNS Microenvironment

The nervous system is a sophisticated cellular network that depends on the delicate equilibrium between glial cells and neurons. Notably, glial cells outnumber neurons by approximately 10 to 1, highlighting their abundance [44]. Glial cells, including microglia and astrocytes, are crucial in establishing and maintaining an optimal environment for neuronal activity. They provide essential structural and metabolic support, regulate the extracellular environment, and facilitate synaptic communication [45]. Without sufficient glial cell support, the neuronal environment can deteriorate, compromising the overall health and functionality of neurons.

In the context of chronic pain, recent studies have emphasized the essential role of glial cells in its pathogenesis and perpetuation [46]. Microglia, the immune cells of the CNS, undergo morphological and functional changes in response to nerve injury, transitioning from a resting to an activated state. When activated, microglia produce inflammatory mediators and growth factors, which leads to increased production of enzymes that generate prostaglandins, ultimately triggering pain signals [47]. Astrocytes, another type of glial cell, often become activated following microglia activation, contributing to a prolonged and persistent pain state [48].

Pregabalin primarily targets neurons, whereas dexborneol primarily acts on glial cells to modulate the neuronal environment (Figure 9), addressing imbalances in neurotransmitters that are often the underlying cause of chronic pain [49]. Its neuroprotective benefits originate from its anti-inflammatory and antioxidative properties, primarily by inhibiting NFκB—a key modulator of inflammation—and activating Nrf2, a transcription factor that encourages the expression of antioxidant genes [50]. These benefits are similar to those offered by natural medicines such as sinomenine and ligustrazine, which also possess neuroprotective qualities, amplifying the analgesic properties of GABAergic anticonvulsants [51]. In clinical settings, the combination of dexborneol and edaravone creates a powerful antioxidative and anti-inflammatory cocktail, positioning it as a potential therapeutic option for treating patients suffering from acute ischemic stroke [52].

In this study, the combined administration of pregabalin and dexborneol effectively suppressed the spinal activation of microglia. As a result, this drug combination reduced the secretion of inflammatory mediators and ATP by neurons and glial cells, ultimately leading to a decrease in neuronal depolarization. Therefore, pregabalin-dexborneol provides a comprehensive treatment approach for inflammation and neuronal depolarization related to nerve damage. This drug combination effectively disrupts two important neuro-immune crosstalk processes. More specifically, it breaks two harmful cycles: one is initiated by HMGB1, which leads to a self-perpetuating cycle of inflammation, and the other is triggered by ATP, resulting in a continuous oxidative cycle (Figure 9).

### 5.4. Pregabalin-Dexborneol Breaks HMGB1-Driven Inflammatory Cycle

Neuroinflammation, triggered by glial cells, stimulates neurons in the CNS, initiating an intricate neuro-immune interaction in chronic pain. In the SNI model, nerve damage and oxidative stress lead to increased expression of inflammatory cytokines, specifically TNF and IL-1β, in the spinal cord. These cytokines then trigger neurons via their receptors, perpetuating a vicious cycle of inflammation and pain.

The combination therapy of pregabalin and dexborneol has demonstrated significant suppression of neuronal/glial NF-κB expression, resulting in minimal release of HMGB1, a potent endogenous activator of TLR4 [49,53]. HMGB1, when released by neurons, interacts with TLR4 receptors on microglia and astrocytes, triggering the release of additional inflammatory mediators such as TNF and IL-1β [54]. This cascade further activates neurons and glial cells, driving a continuous cycle of NF-κB activation and inflammatory mediator release, which in turn stimulates neurons to secrete more HMGB1 [55]. These steps create a positive feedback loop that sustains and exacerbates the inflammatory response. By targeting the HMGB1-TLR4-NFκB pathway, pregabalin-dexborneol offers relief from chronic pain by breaking the vicious cycle of inflammation and neuronal activation (Figure 9).

### 5.5. Nrf2 Activation by Pregabalin-Dexborneol Disrupts a Self-Amplifying Oxidative Cycle

The intricate interplay between neurons and glial cells plays a crucial role in maintaining CNS homeostasis. However, certain pathological conditions can trigger a self-amplifying oxidative cycle, leading to neuronal DNA damage, spinal dorsal horn enhancement, and persistent pain [56]. Oxidative stress and inflammation are closely intertwined processes that can mutually reinforce each other, leading to a self-perpetuating cycle of pro-inflammatory mediator production and, often, pain intensification [57]. 

A crucial component of this cycle is the P2X3 receptor, which is activated by ATP. The ATP-P2X3 inflammatory cycle begins with the neuronal release of ATP, which activates P2X3 receptors located on microglia. This activation triggers the release of inflammatory mediators, including TNF, IL-1β, and IL-6, initiating a cascade of events [58]. These inflammatory mediators, in turn, stimulate astrocytes to release more ATP [59]. This ATP then feeds back to neurons, activating them through neuronal P2X3 receptors and leading to further neuronal release of ATP, perpetuating the cycle of inflammation and oxidative stress (Figure 9).

Fortunately, recent studies have identified Nrf2 as a crucial regulator capable of disrupting the vicious cycle of inflammation and oxidative stress [60]. Nrf2 activation suppresses the inflammatory response and safeguards neurons from oxidative damage [61]. The combined use of pregabalin and dexborneol has been found to effectively increase Nrf2 expression in both neurons and glial cells. This elevation in Nrf2 levels blocks the release of ATP, thereby disrupting the initial step of the ATP-P2X3 inflammatory cycle (Figure 9). This intervention offers a promising therapeutic approach for treating chronic pain conditions linked to neuroinflammation and oxidative stress.

Intriguingly, prolonged and unmanaged oxidative stress can cause severe toxicity to nerve cells, necessitating the timely elimination of the cells generating such oxidative stress. The combination of pregabalin and dexborneol has exhibited significant anti-oxidative properties. However, further research is required to determine whether their neuroprotective effects arise from inducing apoptosis or pyroptosis.

### 5.6. Lipid Metabolism as Key Upstream Mechanism Driving Pregabalin-Dexborneol’s Neuroprotection

Lipids are crucial components of life processes, performing essential biological functions. The mechanisms of some drugs are tied to lipid regulation, as exemplified by aspirin’s key role in regulating endogenous lipid synthesis for its therapeutic effect [62]. Despite this, the complex and diverse nature of lipid molecules had hindered research progress in lipid metabolism and functional regulation over a long period. Recently, the application of soft ionization and high-resolution mass spectrometry techniques in lipid analysis has facilitated a comprehensive and systematic understanding of lipids and their interactions in organisms, tissues, or cells, giving rise to the emerging field of lipidomics [62].

Subsequently, lipidomics research exploring the connection between lipid metabolism and pain has gained increasing attention, emerging as a new frontier in pain studies. As an example, Patti et al. employed an untargeted lipidomics method and uncovered that nerve injury can trigger alterations in sphingomyelin-ceramide metabolism within the central nervous system [63]. This, in turn, leads to an increase in the lipid levels of N,N-dimethylsphingosine (DMS). Additionally, through the direct infusion of DMS into the spinal canal, it was established that DMS plays a significant role in the onset of neuropathic pain [64].

PCs, renowned for their anti-inflammatory, anti-oxidative, and neuronal protective qualities, seem to be pivotal in mitigating inflammatory and oxidative reactions [65]. In the present study, by utilizing the cutting-edge technology of lipidomics, we identified elevated levels of PCs in the spinal cord of animals with SNI-induced chronic pain, following the treatment with pregabalin-dexborneol. The observed accumulation of PCs might underlie pregabalin-dexborneol’s neuroprotective effects in pain scenarios, serving as a potential upstream mechanism (Figure 9). Although our initial findings are promising, further investigations are necessary to gain a deeper understanding of the precise mechanisms by which PCs exert their therapeutic effects.

## 6. Conclusions

Pregabalin, on its own, has limitations such as ineffectiveness and dose-dependent adverse reactions. However, dexborneol enhances pregabalin’s efficacy by promoting its transformation, disrupting two self-amplifying vicious cycles by decreasing HMGB1 and ATP release, and exerting profound anti-oxidative effects through lipid modulation. This reduces general neuroinflammation, addressing the root causes of chronic pain. By amplifying pregabalin’s analgesic properties and allowing for reduced doses, this drug combination minimizes adverse systemic reactions and side effects. This breakthrough paves the way for more effective and tolerable pain management options, prioritizing both efficacy and safety.

## Figures and Tables

**Table 1:** The inhibition ratio (%) and Jong-kyun’s value.

Models and Pains	Pregabalin 50 mg/kg	Dexborneol 300 mg/kg	Pregabalin 50 mg/kg + Dexborneol 300 mg/kg	Jong-Kyun’s q Value
mechanical pain of SNI	13.95	−0.44	37.91	2.79
cold pain of SNI	14.73	−3.12	43.30	3.59
mechanical pain of INCI	6.32	−0.24	21.54	3.54
cold pain of INCI	14.41	6.80	26.69	1.32

**Table 2:** Effects of dexborneol on the pharmacokinetic parameters of pregabalin in blood and brain tissue. Caption: The table presents key pharmacokinetic parameters for pregabalin in blood and brain tissue, with and without the administration of dexborneol. AUC (0–t) and AUC (0–∞) represent the total exposure to the drug over time, from the initial administration to the last measurable concentration and extrapolated to infinity, respectively. MRT (0–t) and MRT (0–∞) indicate the average time a drug molecule spends in the body. The terminal half-life (t1/2z) shows how long it takes for the drug concentration to halve during the final elimination phase. Tmax denotes the time it takes to reach the peak drug concentration (C_max_) in plasma or brain tissue. Vz/F estimates the drug’s distribution volume adjusted for bioavailability, while CLz/F represents clearance adjusted for bioavailability, indicating the rate of drug removal from the plasma.

		Plasma		Brain	
Parameter	Units	Pregabalin 50 mg/kg	Pregabalin 50 mg/kg + Dexborneol 300 mg/kg	Pregabalin 50 mg/kg	Pregabalin 50 mg/kg + Dexborneol 300 mg/kg
AUC (0–t)	μg/L·h	111,623.20 ± 7951.00	131,453.10 ± 14,515.35	16,913.72 ± 1675.77	21,313.56 ± 3665.82
AUC (0–∞)	μg/L·h	111,921.78 ± 7824.53	131,821.18 ± 14,443.95	17,763.44 ± 1819.73	22,617.36 ± 4462.52
MRT (0–t)	h	1.80 ± 0.22	1.96 ± 0.22	2.88 ± 0.24	3.09 ± 0.19
MRT (0–∞)	h	1.82 ± 0.22	1.99 ± 0.21	3.24 ± 0.32	3.49 ± 0.64
t1/2z	h	0.84 ± 0.10	0.86 ± 0.12	1.60 ± 0.27	1.49 ± 0.60
T_max_	h	0.58 ± 0.20	0.83 ± 0.61	1.17 ± 0.41	2.17 ± 0.41
Vz/F	L/kg	0.54 ± 0.10	0.48 ± 0.10	6.48 ± 0.94	4.74 ± 1.45
CLz/F	L/h/kg	0.45 ± 0.03	0.38 ± 0.04	2.84 ± 0.27	2.28 ± 0.45
C_max_	μg/L	56,254.25 ± 8451.01	60,860.60 ± 13,515.34	4288.62 ± 450.78	5581.82 ± 1368.57

**Table 3:** Lipids contributing to the analgesic synergy of pregabalin and dexborneol (FC: fold change). The structures of all 10 lipids can be found in the Supplementary Material (Supporting Information—Structures of Lipids in Table 3).

Compounds	Molecular Formula	ANOVA	ANOVA	FC
		(Inter Groups)	(PB vs. P)	(PB vs. P)
PC(16:0/22:4(7Z,10Z,13Z,16Z))	C_46_H_84_NO_8_P	2.45 × 10^−4^	3.34 × 10^−3^	4.94
PC(15:1(9Z)/22:6(4Z,7Z,10Z,13Z,16Z,19Z))	C_45_H_76_NO_8_P	5.28 × 10^−5^	4.17 × 10^−4^	3.54
PC(17:1(9Z)/22:6(4Z,7Z,10Z,13Z,16Z,19Z))	C_47_H_80_NO_8_P	1.07 × 10^−4^	1.99 × 10^−3^	4.02
PC(13:0/18:2(9Z,12Z))	C_39_H_74_NO_8_P	9.63 × 10^−6^	6.03 × 10^−4^	3.15
PC(16:1(9Z)/22:6(4Z,7Z,10Z,13Z,16Z,19Z))	C_46_H_78_NO_8_P	7.94 × 10^−1^	9.85 × 10^−1^	1.45
SM(d18:1/16:0)	C_39_H_79_N_2_O_6_P	5.22 × 10^−6^	7.15 × 10^−4^	3.63
DG(20:5(5Z,8Z,11Z,14Z,17Z)/20:5(5Z,8Z,11Z,14Z,17Z)/0:0)	C_43_H_64_O_5_	5.37 × 10^−3^	6.79 × 10^−2^	38.54
DG(20:0/22:3(10Z,13Z,16Z)/0:0)	C_45_H_82_O_5_	1.64 × 10^−3^	5.54 × 10^−2^	8.29
PS(13:0/18:3(6Z,9Z,12Z))	C_37_H_66_NO_10_P	4.96 × 10^−3^	8.15 × 10^−2^	2.96
MG(20:0/0:0/0:0)	C_23_H_46_O_4_	4.28 × 10^−5^	2.66 × 10^−4^	4.25

## Data Availability

The dataset presented in this article is available upon request. Those interested in accessing the raw data that underlies the findings and conclusions of this study may contact the authors for availability.

## Supplementary Materials

The following supporting information can be downloaded at: https://www.mdpi.com/article/10.3390/antiox13070803/s1.

## Abbreviations

CDC	Centers for Disease Control and Prevention
GABA	γ-aminobutyric acid
ROS	reactive oxygen species
SOD	superoxide dismutase
ATP	adenosine triphosphate
MDA	malondialdehyde
LMs	lipid mediators
C	control group
P	pregabalin group
PB	combination of pregabalin and dexborneol group
B	dexborneol group
iNOS	inducible nitric oxide synthase
GFAP	glial fibrillary acidic protein
PC	phosphatidylcholine
DHA	docosahexaenoic acid
LAT1	L-type Amino Acid Transporter 1
NMDA	N-Methyl-D-aspartic acid
AMPA	α-amino-3-hydroxy-5-methyl-4-isoxazolepropionic acid
DMS	N,N-dimethylsphingosine
