# Supplementary material for: Dexborneol Amplifies Pregabalin’s Analgesic Effect in Mouse Models of Peripheral Nerve Injury and Incisional Pain
Source: Antioxidants (Basel). 2024 Jul 2;13(7):803. doi: 10.3390/antiox13070803 (PMC11273404; doi:10.3390/antiox13070803)

Supporting Information-Structures of Lipids in Table 3

1. PC (16:0/22:4(7Z,10Z,13Z,16Z))

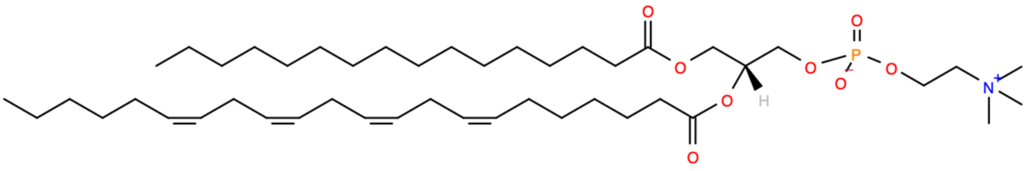

2. PC (15:1(9Z)/22:6(4Z,7Z,10Z,13Z,16Z,19Z))

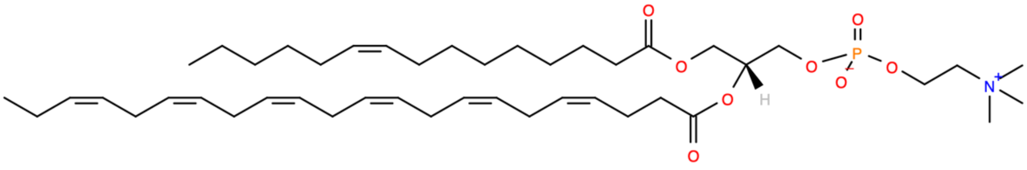

3. PC (17:1(9Z)/22:6(4Z,7Z,10Z,13Z,16Z,19Z))

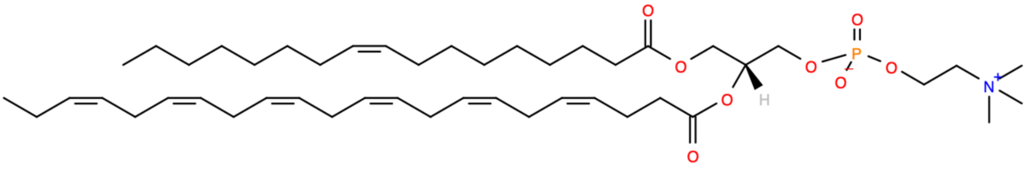

4. PC (13:0/18:2(9Z,12Z))

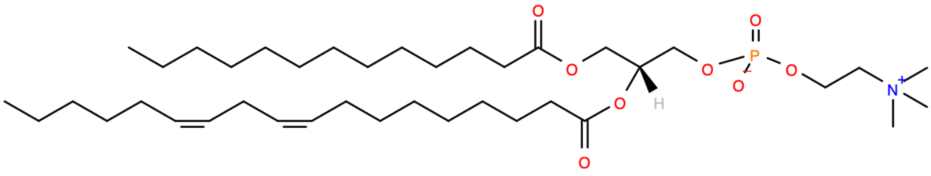

5. PC (16:1(9Z)/22:6(4Z,7Z,10Z,13Z,16Z,19Z))

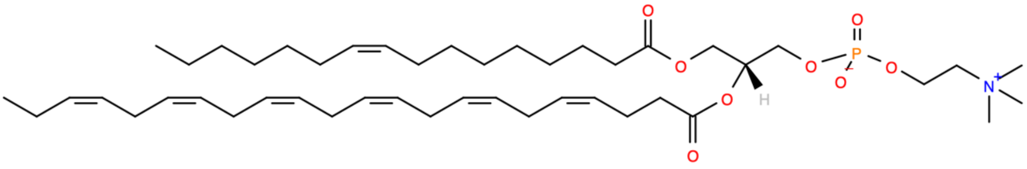

6. SM (d18:1/16:0)

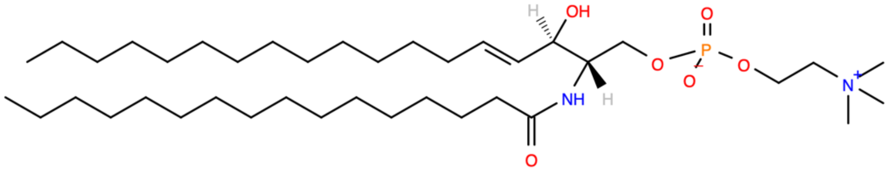

7. DG (20:5(5Z,8Z,11Z,14Z,17Z)/20:5(5Z,8Z,11Z,14Z,17Z)/0:0)

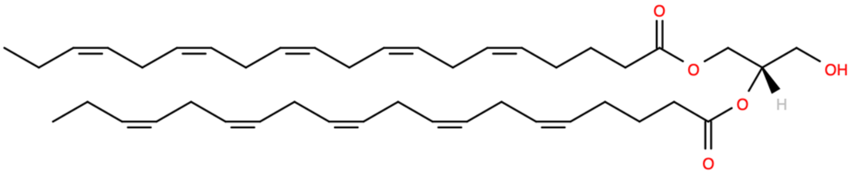

8. DG (20:0/22:3(10Z,13Z,16Z)/0:0)

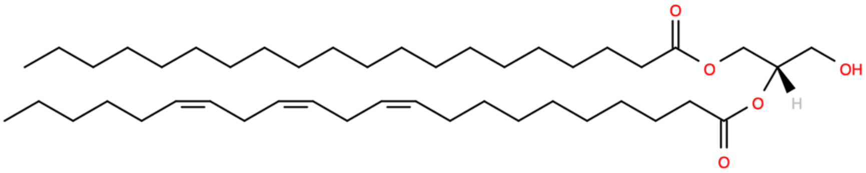

9. PS (13:0/18:3(6Z,9Z,12Z))

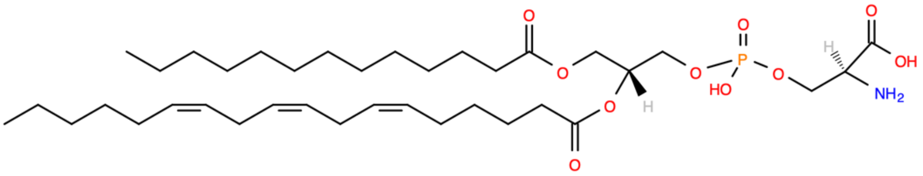

10. MG (20:0/0:0/0:0)

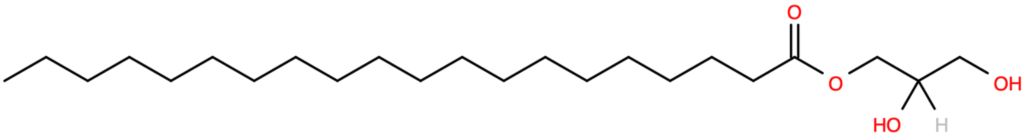

Supplement: Supplementary file 1 [file antioxidants-13-00803-s001.zip › antioxidants-3078015-supplementary.pdf]
